# Poly(β-Amino Ester)-Nanoparticle Mediated Transfection of Retinal Pigment Epithelial Cells *In Vitro* and *In Vivo*

**DOI:** 10.1371/journal.pone.0037543

**Published:** 2012-05-21

**Authors:** Joel C. Sunshine, Sarah B. Sunshine, Imran Bhutto, James T. Handa, Jordan J. Green

**Affiliations:** 1 Biomedical Engineering, Johns Hopkins University School of Medicine, Baltimore, Maryland, United States of America; 2 Wilmer Eye Institute, Johns Hopkins University School of Medicine, Baltimore, Maryland, United States of America; Johns Hopkins School of Medicine, United States of America

## Abstract

A variety of genetic diseases in the retina, including retinitis pigmentosa and leber congenital amaurosis, might be excellent targets for gene delivery as treatment. A major challenge in non-viral gene delivery remains finding a safe and effective delivery system. Poly(beta-amino ester)s (PBAEs) have shown great potential as gene delivery reagents because they are easily synthesized and they transfect a wide variety of cell types with high efficacy *in vitro*. We synthesized a combinatorial library of PBAEs and evaluated them for transfection efficacy and toxicity in retinal pigment epithelial (ARPE-19) cells to identify lead polymer structures and transfection formulations. Our optimal polymer (B5-S5-E7 at 60 w/w polymer∶DNA ratio) transfected ARPE-19 cells with 44±5% transfection efficacy, significantly higher than with optimized formulations of leading commercially available reagents Lipofectamine 2000 (26±7%) and X-tremeGENE HP DNA (22±6%); (p<0.001 for both). Ten formulations exceeded 30% transfection efficacy. This high non-viral efficacy was achieved with comparable cytotoxicity (23±6%) to controls; optimized formulations of Lipofectamine 2000 and X-tremeGENE HP DNA showed 15±3% and 32±9% toxicity respectively (p>0.05 for both). Our optimal polymer was also significantly better than a gold standard polymeric transfection reagent, branched 25 kDa polyethyleneimine (PEI), which achieved only 8±1% transfection efficacy with 25±6% cytotoxicity. Subretinal injections using lyophilized GFP-PBAE nanoparticles resulted in 1.1±1×10^3^-fold and 1.5±0.7×10^3^-fold increased GFP expression in the retinal pigment epithelium (RPE)/choroid and neural retina respectively, compared to injection of DNA alone (p = 0.003 for RPE/choroid, p<0.001 for neural retina). The successful transfection of the RPE *in vivo* suggests that these nanoparticles could be used to study a number of genetic diseases in the laboratory with the potential to treat debilitating eye diseases.

## Introduction

Many of the most debilitating ocular diseases are caused by gene deletion mutations. The ability of an ocular disease to be treated with a single gene replacement therapy was shown to be successful in principle in a canine model of Leber's Congenital Amaurosis in which RPE 65 was replaced using a recombinant adeno associated viral (AAV) delivery system that resulted in visual restoration in these animals [1]. Furthermore, early phase clinical trials studying this therapy in people suggest that gene therapy is a feasible potential strategy for retinal dystrophies [2]. A variety of genetic diseases of the retina, including retinitis pigmentosa, Best's disease, and Stargardt's disease might be excellent targets for gene replacement therapy.

Despite the initial success of gene replacement, gene delivery methods need to be optimized. There are significant limitations to viral delivery systems such as the AAV vector. The AAV vector is limited in what ocular diseases it could potentially treat because it is capable of optimally carrying 4.7–4.9 kilobases (kb) with a maximum carrying capacity of 5.2 kb [3] while many ocular diseases are caused by mutations to genes that are larger than 5 kb. Notable examples include Best's disease, caused by a mutation in Bestrophin-1 (14.6 kb) [4], or Stargardt's disease, caused by a mutation in the ABCA4 gene (6.8 kb) [5]. To address this problem, we recently reported that our non-viral delivery system using poly(beta-amino) esters (PBAEs) can accommodate large inserts to deliver up to 100 plasmids and ∼500 kbp of nucleic acid per nanoparticle [6].

Non-viral systems for gene delivery offer a host of potential advantages over viruses, including reduced toxicity [7], reduced immunogenicity [8], and ease of production. However, to date, most non-viral systems are significantly less efficient at transfecting hard to transfect cell types compared to viral methods, so the major challenge remains finding an effective non-viral delivery system [9], [10]. Multiple alternative non-viral gene delivery approaches have been reported in the literature for *in vitro* transfection of retinal pigment epithelial (RPE) cells, with limited success. One report on transfection of the established RPE cell line, ARPE-19 cells, with solid lipid nanoparticles details difficulty in uptake of the particles that resulted in a transfection efficacy of only 2.5% [11]. Another study looked at optimizing several different commercially available reagents (Tfx-50, Lipofectin, Lipofectamine, Cellfectin, and DMRIE-C) for the transfection of primary RPE cells [12]. This study utilized serum free-conditions and non-confluent RPE cultures, which are associated with higher transfection efficacy [13], yet the best reagent formulated optimally, Tfx-50 at 3∶1 DNA∶liposome ratio, only achieved 12–15% transfection efficiency [12]. Another study looking at the transfection of primary RPE cells showed transfection efficiencies of less than 1% for Lipofectin, between 1 and 3% for DOTAP/DOGS, and up to 5% by using degraded dendrimers [13]. This limited success in transfection of ARPE-19 cells or primary RPE cells *in vitro* indicates a need for improved transfection reagents for use in the lab.

**Figure 1:**
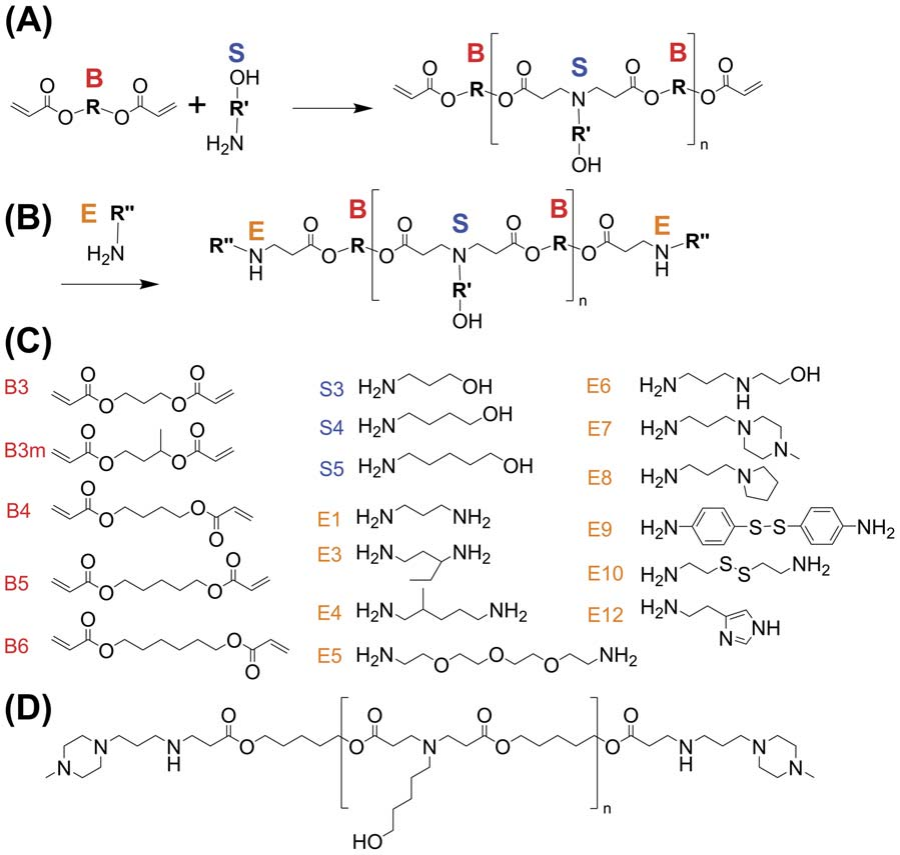
Schematic showing polymerization scheme and monomers used. (A) Diacrylates (“B”) were added to primary-amine containing amino-alcohol side chains (“S”) to form the base polymers. (B) Base polymers were end-capped with amine monomers (“E”) to form the final, end-modified polymers. (C) The base diacrylate (“B”), amino-alcohol side chain (“S”), and end-modifying amines (“E”) used in the polymer library are listed here. (D) The full structure of B5-S5-E7 (1-(3-aminopropyl)-4-methylpiperazine-end-modified poly(1,5 pentanediol diacrylate-co-5-amino-1-pentanol) is shown here.

**Figure 2:**
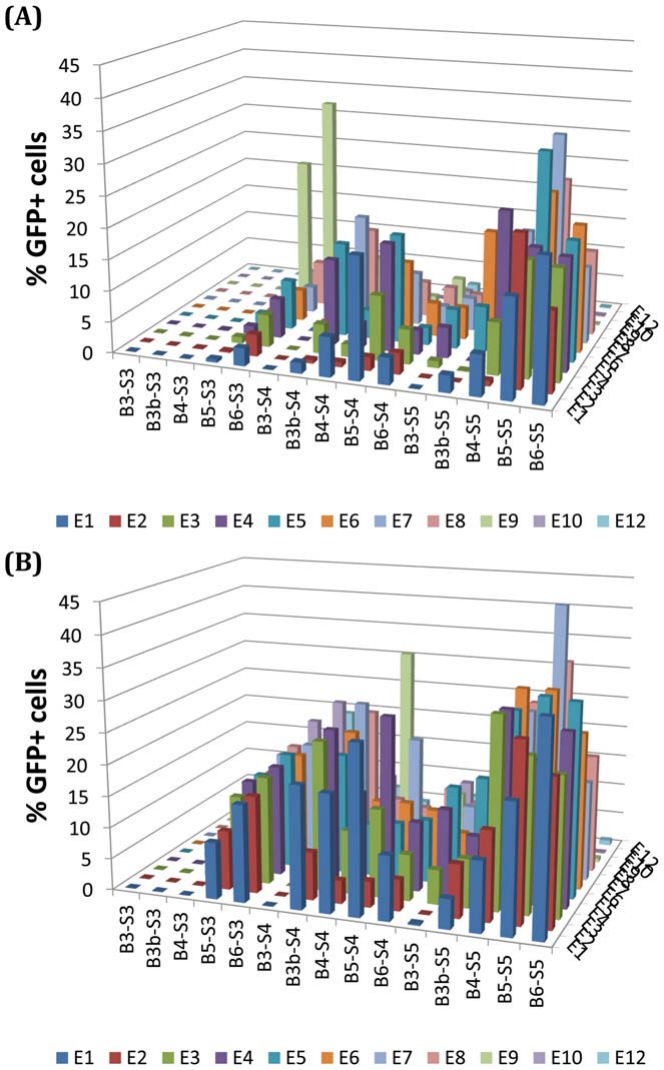
Bar graph displaying confluent ARPE-19 cell-sheet transfection efficacy (%GFP+ cells by FACS) of polymer formulations (n = 4) in our library screen. (A) Transfection efficacy of polymer library formulated at 30 w/w ratio. (B) Transfection efficacy of polymer library formulated at 60 w/w ratio. Optimal formulation B5-S5-E7 at 60 w/w resulted in 44% transfection efficacy as compared to 26% for Lipofectamine 2000, 22% for ExtremeGENE HP DNA, and 8% for branched 25 kDa PEI.

**Figure 3 pone-0037543-g003:**
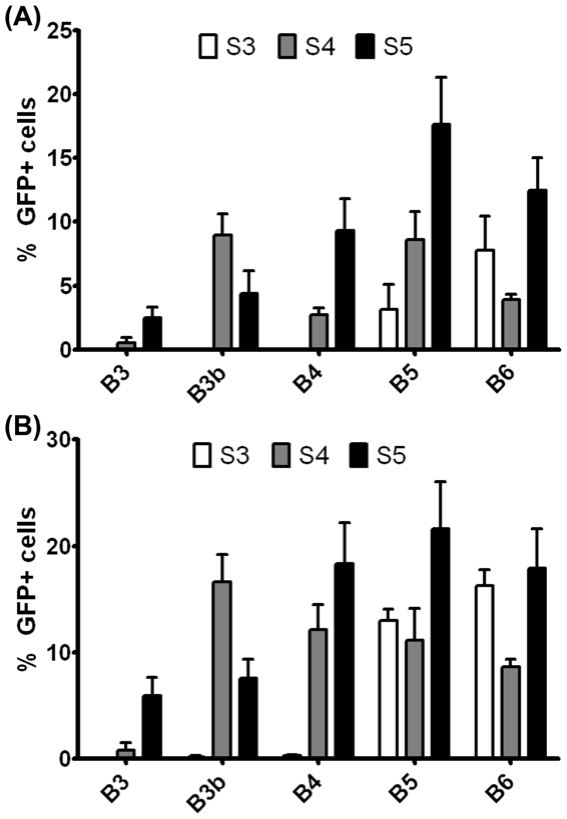
Comparison of base polymer structure with transfection efficacy. Each bar represents the average transfection efficacy associated with the end-modified polymers that contained the base polymer shown (n = 11; error bar = SEM). Base diacrylate and side chain amino-alcohols are shown from least hydrophobic to most hydrophobic from left to right. (A) Transfection efficacy of 30 w/w formulations averaged over 11 end-modified amines containing the base polymer shown. (B) Transfection efficacy of 60 w/w formulations averaged over 11 end-modified amines containing the base polymer shown. For statistical analysis, see Table 1.

**Table 1:** Results of 2-way ANOVA examining the effect of increased hydrophobicity of the side chain with respect to the base diacrylate it is paired with.

Reduction in Metabolic Activity				Transfection Efficacy			
30 w/w	S3->S4	S4->S5	S3->S5	30 w/w	S3->S4	S4->S5	S3->S5
B3	NS	NS	NS	B3	NS	NS	NS
B3b	NS	NS	NS	B3b	**	NS	NS
B4	NS	*	**	B4	NS	NS	**
B5	***	NS	***	B5	NS	**	***
B6	NS	**	**	B6	NS	**	NS

60 w/w	S3->S4	S4->S5	S3->S5	60 w/w	S3->S4	S4->S5	S3->S5
B3	NS	***	***	B3	NS	NS	NS
B3b	NS	*	NS	B3b	***	NS	NS
B4	**	NS	***	B4	*	NS	***
B5	**	NS	***	B5	NS	**	*
B6	***	NS	***	B6	NS	*	NS

NS is non-significant; P<0.05 is *; P<0.01 is **; P<0.001 is ***.

Poly(beta-amino) esters (PBAEs) [14] have shown great potential as gene delivery reagents and are easily synthesized, rapidly screened, and can be transfected into a wide variety of cell types with high efficacy *in vitro* [15], [16], [17], [18], [19]. PBAE nanoparticles have several advantages which help to overcome the barriers to intracellular plasmid DNA (pDNA) delivery [10]. PBAEs, when added to pH 5 buffer, are positively charged and can spontaneously form positively-charged nanoparticles (generally less than 200 nm) when added to negatively charged pDNA [6]. They get taken up via endocytosis, and enable endosomal escape by buffering the endosome [20]. They are degraded by hydrolysis of the ester bonds in the polymer backbone, enabling reduced cytotoxicity when compared to non-degradable controls [14]. Previous studies have indicated that not only was the base polymer important to its gene delivery properties, but that modification of the polymer ends can further improve transfection efficiency [15], [16], [17], [20]. We have recently found that PBAEs are highly effective (65%–85%) for the transfection of human macrovascular cells (human umbilical vein endothelial cells) and human microvascular cells (human retinal endothelial cells) and that although the efficacies of these nanoparticles are highly correlated between human vascular cell types, their efficacies are uncorrelated to human retinal pigment epithelial cells, which are also a more difficult cell type to transfect [21].

**Figure 4 pone-0037543-g004:**
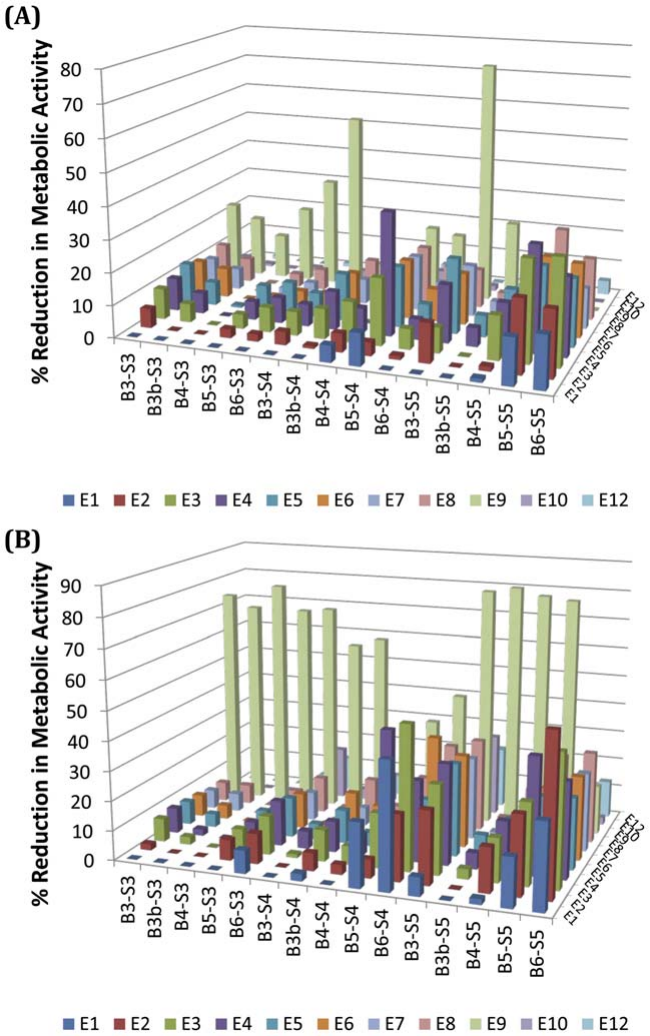
Reduction in metabolic activity following PBAE nanoparticle administration. Formulations plotted at 0% reduction of metabolic activity here had equivalent or slightly higher metabolic activity than untreated controls. (A) Reduction in metabolic activity post transfection with polymer library formulated at 30 w/w ratio (n = 4). (B) Reduction in metabolic activity post transfection with polymer library formulated at 60 w/w ratio (n = 4).

**Figure 5 pone-0037543-g005:**
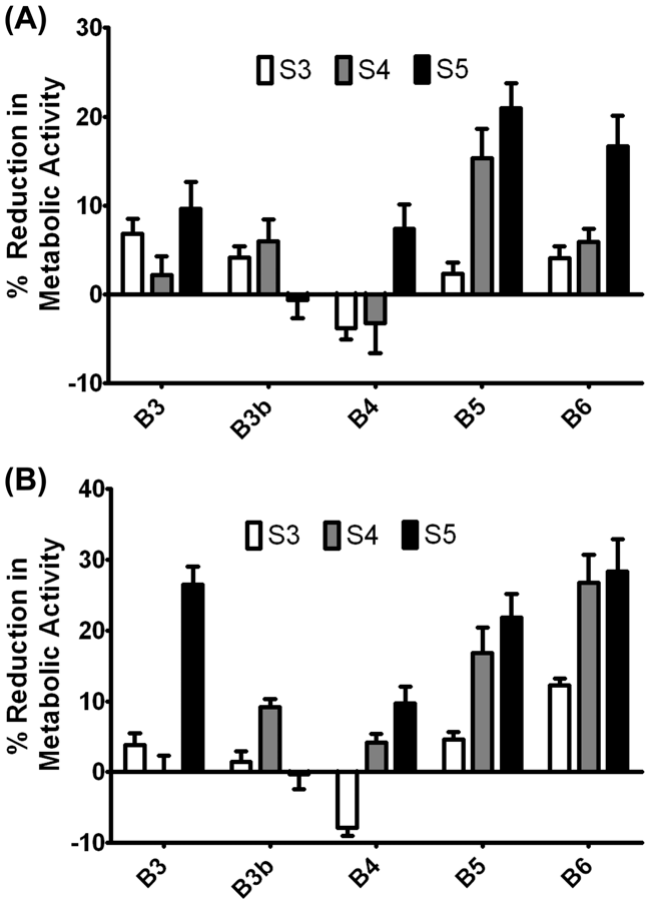
Comparison of base polymer structure with reduction in metabolic activity. Each bar represents the average toxicity associated with the end-modified polymers that contained the base polymer shown (n = 11; error bar = SEM). Base diacrylate and side chain amino-alcohols are shown from least hydrophobic to most hydrophobic from left to right. (A) Reduction in metabolic activity of 30 w/w formulations averaged over 10 end-modified amines containing the base polymer shown. (B) Reduction in metabolic activity of 60 w/w formulations averaged over 10 end-modified amines containing the base polymer shown. For statistical analysis, see Table 1.

**Figure 6:**
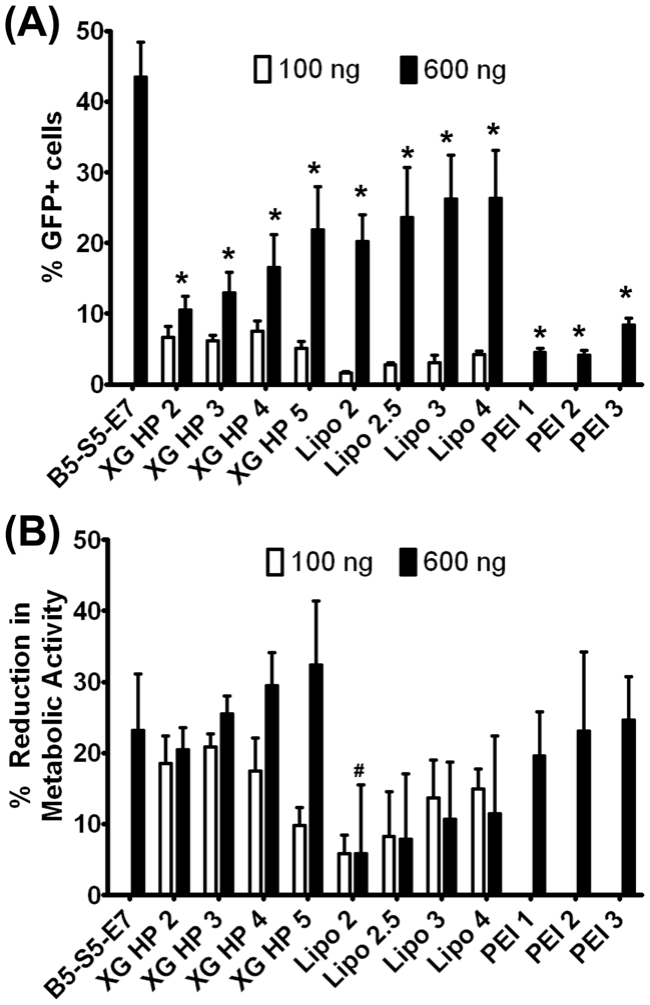
Comparison to commercially available transfection reagents. (XG HP = XtremeGENE HP; Lipo = Lipofectamine 2000; PEI = 25 kDa branched polyethyeleneimine). The numbers next to the reagent name corresponds to the ratio of lipid (v/v) or polymer (w/w) to DNA used. (A) Transfection efficacy of control formulations and B5-S5-E7 at 60 w/w (n = 4); * indicates p<0.001 vs. B5-S5-E7 at 60 w/w. (B) Reduction of metabolic activity of control formulations and B5-S5-E7 at 60 w/w (n = 4); # indicates p<0.05 vs. B5-S5-E7 at 60 w/w.

**Figure 7:**
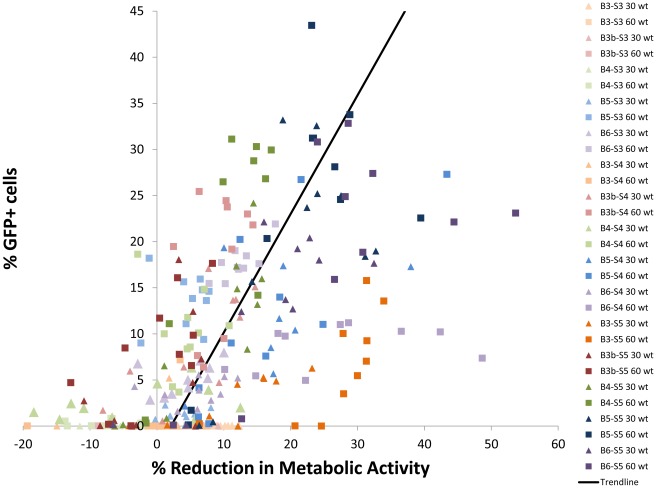
Comparison of transfection efficacy to reduction in metabolic activity of the polymers in the polymer library. There is an overall trend of increasing cytotoxicity with increasing transfection efficiency (the best-fit line represents a 0.77% decrease in cell metabolic activity with every 1% increase in transfection efficiency of the formulation) but the trend only explains a portion of the results (R^2^ = 0.37). A number of polymer formulations exhibited high transfection efficiencies and low concomitant cytotoxicities.

In this study, we synthesized and evaluated an expanded combinatorial library of PBAEs for evaluation of transfection efficacy and toxicity in ARPE-19 cells to identify lead polymer structures and transfection formulations for this difficult-to-transfect cell type. We discovered a lead polymer, polymer (1-(3-aminopropyl)-4-methylpiperazine-end-modified poly(1,5 pentanediol diacrylate-co-5-amino-1-pentanol) (B5-S5-E7) and characterized it for the first time in terms of polymer molecular weight, polymer half-life, nanoparticle size, nanoparticle zeta potential, and vitro efficacy compared to leading commercially available reagents. To validate the *in vitro* screen and to evaluate the efficacy of a PBAE-based nanoparticle for gene delivery to the eye for the first time, nanoparticles were administered to mouse RPE *in vivo* by subretinal injection. Lyophilized DNA nanoparticles, utilizing a technology designed to enhance the stability and shelf-life of the biodegradable nanoparticles, were used for the *in vivo* injections. These novel nanoparticles offer opportunity both for studying diseases in the laboratory and as a potential treatment modality.

**Figure 8:**
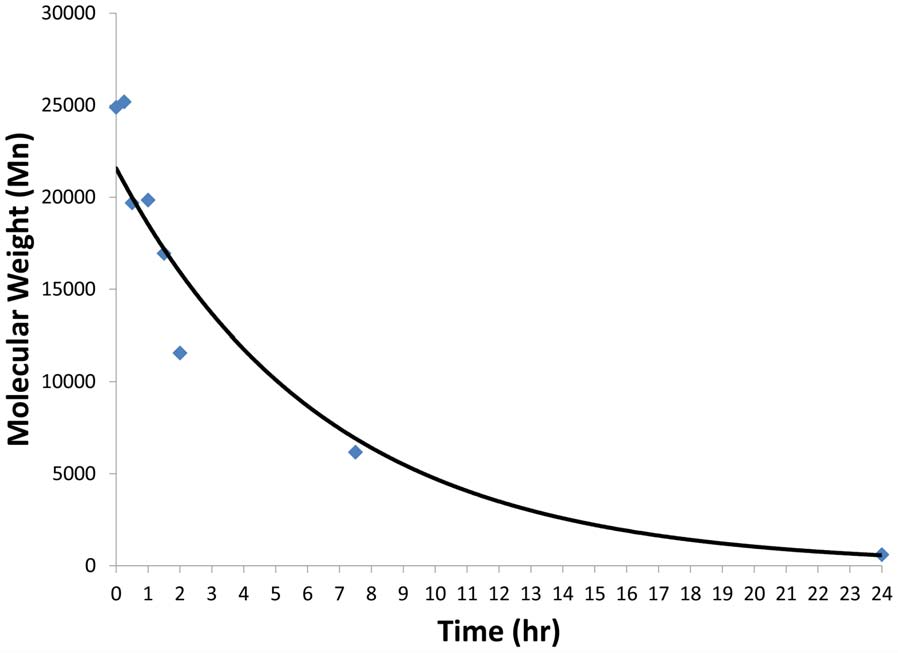
Number-averaged molecular weight versus time of B5-S5-E7 in PBS at 37°C with agitation. The half-life of the polymer in solution was 4.6 hr (R^2^ = 0.984), and the polymer was almost completely degraded within 1 day.

**Figure 9:**
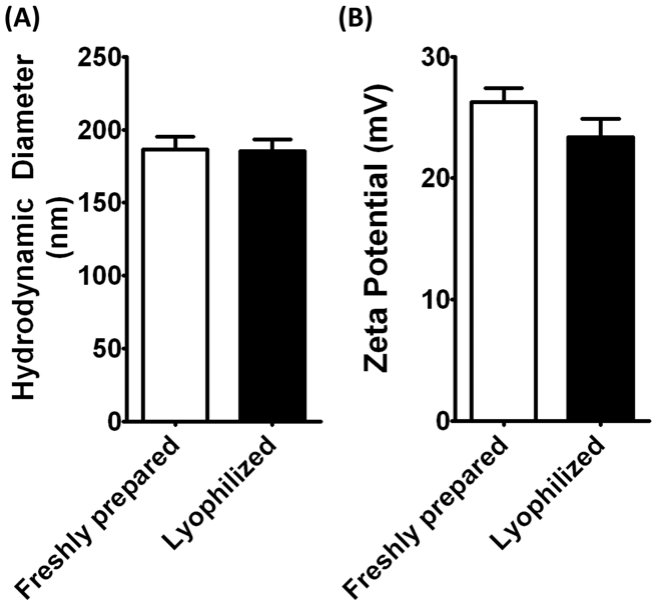
Biophysical characterization of nanoparticles before and after lyophilization. (A) Hydrodynamic diameter of freshly prepared DNA/B5-S5-E7 particles versus lyophilized particles (n = 3; bars are standard error). (B) Zeta potential of freshly prepared DNA/B5-S5-E7 particles versus lyophilized particles (n = 3; bars are standard error). Differences in particle size (p = 1) and zeta potential (p = 0.05) are not significant.

**Figure 10:**
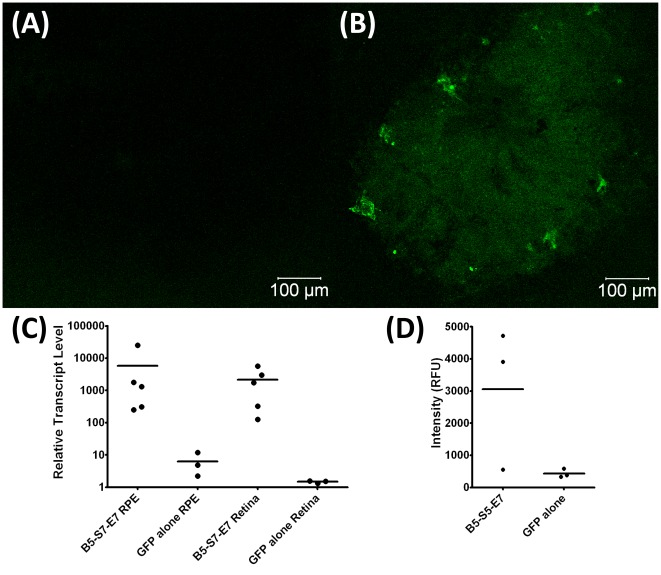
Confocal image of RPE/choroid flat mount post subretinal injections; green corresponds to fluorescence due to GFP expression. Both images were taken with the same camera settings. (A) pDNA alone. (B) pDNA/nanoparticle injection. (C) Relative transcript level to GAPDH (set at 10,000) of GFP mRNA expression after subretinal injection of PBAE eGFP nanoparticles and subretinal injection of naked DNA. Each injection is diplayed as a separate point, and the mean relative transcript level is displayed as a bar. Subretinal injections using lyophilized GFP-PBAE nanoparticles resulted in 1.1±1×10^3^-fold and 1.5±0.7×10^3^-fold increased GFP expression in the RPE/choroid and neural retina, respectively, compared to injection of DNA alone (p = 0.003 for RPE/choroid, p<0.001 for neural retina). (D) Relative fluorescence intensity of retinal flat mounts after subretinal injection with GFP nanoparticles and GFP plasmid alone (n = 3).

## Materials and Methods

### Materials

All reagents and solvents were obtained from commercial suppliers and used as received. Monomers were purchased from Acros Organics, Alfa Aesar, Fluka, Monomer-Polymer and Dajac Laboratories, Sigma-Aldrich, and TCI America. CMV-eGFP was amplified by Aldevron (Fargo, ND). X-tremeGENE HP DNA, Lipofectamine 2000, and Opti-MEM I were purchased from Invitrogen and used according to the manufacturer's instructions. 25 kDa PEI was purchase from Sigma Aldrich and diluted to a stock solution at 1 mg/ml in deionized water. 96 AQ_ueous_ One MTS assay was purchased from Promega and used according to the manufacturer's instructions. Dulbecco's Modified Eagle Medium (DMEM) F12 was purchased from Invitrogen and supplemented with 10% fetal bovine serum (FBS; Invitrogen).

### Polymer Synthesis

End-modified PBAEs were synthesized in a two-step process, first by synthesizing the base polymer via Michael addition of primary amines to diacrylates (**Figure 1A**). The library of PBAEs was synthesized by adding primary amines to diacrylate compounds (1.2∶1 molar ratio of diacrylate∶amine) at 90°C for 24 h (**Figure 1A**). The base polymerization reaction was performed without the use of any solvent in 20 mL glass scintillation vials, in an oven in the dark under magnetic stirring. By providing excess diacrylate in the initial polymerization reaction, diacrylate-terminated polymer was synthesized. We have recently described the synthesis and characterization of these materials [17]. The naming convention used here is designed to describe the chemical structure of each polymer. The diacrylates form the base (“B”) chain of the polymer, and the primary amines form the side (“S”) chains of the forming polymer. To further identify the structure of the base or side chain, a number corresponding to the number of carbons in the hydrocarbon portion of the “B” or “S” is appended; thus, B5 is a base diacrylate with 5 carbons in it's hydrocarbon portion between acrylate groups, and B5-S5 is a base polymer with 5 carbons in the hydrocarbon portion of its side chain between the amine and alcohol groups as well as 5 carbons in its hydrocarbon portion of its base chain between acrylate groups. In a second step, the acrylate ends of the polymer intermediates are modified by a second amine, resulting in the final end-modified polymers (**Figure 1B**). The base polymers were end-capped by end-capping (“E”) amines (at 10-fold molar excess of amine to diacrylate termini) at room temperature in DMSO at 100 mg/ml for 1 hr (**Figure 1B**). Specifically, 80 mg of base polymer was dissolved in 400 µl of DMSO, and combined with 320 µl of a 0.5 M solution in DMSO of the end-capping amine, and placed on a multitube vortexer (VWR) and vortexed for 1 hr at 1000 RPM. The library of monomers used here is shown (**Figure 1C**). For the end capping amines, the number is simply sequential; B5-S5-E7 is an end-modified polymer with 5 carbons in the base, 5 carbons on the side chain, and which was end-modified with the E7 end-capping amine. For this study we synthesized a combinatorial library of PBAEs using 5 diacrylates, 3 amino-alcohol side chains, and 11 end-modifying amines to form 165 end-modified polymers with only small, single carbon changes to the backbone and side chains, and small modifications at the ends of the polymers. Base polymerization of all base polymers and end-capping of one base-polymer with all end-capping amines was verified by NMR; for spectra, see [17].

### In vitro Polymer Library and Commercial Control Transfection Screening

ARPE-19 cells were maintained as previously described [22], plated at 100,000 cells/cm^2^ in 96-well plates, and allowed to grow for 72 hr until visually confluent prior to transfection with nanoparticles containing eGFP pDNA. To form the particles, polymer stock solutions at 100 mg/ml in DMSO and pDNA stock solutions at 1 mg/ml in water were separately dissolved in 25 mM sodium acetate (NaAc) buffer at pH 5.0, then combined and mixed by pipeting. Ten minutes later, 20 µl of particle solution containing 600 ng pDNA and 36 µg polymer was added to 100 µl of medium on the cells and allowed to incubate for 4 hr, when the medium was then replaced with fresh medium. For the commercially available lipid based controls Lipofectamine 2000 and X-tremeGENE HP DNA, reagents were formulated as specified by the company (at 100 ng pDNA/well), and also tested at the same DNA dose as used in our screening (600 ng/well) to ensure that optimal, comparable controls were chosen. For PEI transfection, a PEI stock solution at 1 mg/ml was diluted in Opti-MEM I, and mixed with pDNA in Opti-MEM. The solution was then vortexed and allowed to stand for 20 minutes before being added to cells. 24 hr post-transfection, cell viability was analyzed by the CellTiter 96® AQ_ueous_ One MTS assay using a plate reader (BioTek® Synergy 2), and is reported as a reduction in cell viability relative to untreated wells (100% – absorbance of well/untreated, normalized to no cells in the well). 48 hr post-transfection, transfection efficacy was analyzed by flow cytometry (Accuri C6 with Hypercyte high-throughput plate adaptor).

### Preparation of lyophilized nanoparticles

Freeze dried pDNA nanoparticles were prepared by dissolving 36 µl of 100 mg/ml polymer in DMSO in 324 µl of 25 mM sodium acetate buffer (pH 5.0), and then adding 60 µl of 1 mg/ml pDNA (total 60 µg) with 60 µl of 25 mM sodium acetate buffer (pH 5.0). The mixture was vortexed, and allowed to self-assemble for 10 minutes. The nanoparticles were split into two batches and 120 µl of 90 mg/ml sucrose was added to each. The sample was then flash-frozen in liquid nitrogen, and placed on a lyophilizer for freeze-drying. The dried particles (containing 30 µl pDNA) were then resuspended to 30 µl total volume in deionized water (thus resulting in a particle DNA concentration of 1 mg/ml) just prior to subretinal injection.

### Particle Sizing and Zeta Potential measurements

Particle sizing was performed using a NanoSight NS500 (NanoSight Ltd. Wiltshire, UK). Each sample was diluted 1∶50 from the transfection concentrations (n = 3); for the lyophilized samples, each sample was diluted to the same DNA concentration as the freshly prepared samples. For zeta potential measurements, 800 microliters of particle solution containing 5 micrograms of pDNA was diluted into PBS to a total volume of 800 µl and added to a disposable zeta cuvette, and measured in using a Malvern Zetasizer NanoZS.

### Polymer Degradation

250 µl of a 100 mg/ml solution of B5-S5-E7 in DMSO was added to 250 mL of phosphate buffered saline (PBS) solution at 37°C. At each time point, 25 mL of this solution was removed and frozen, then lyophilized to remove the water. This sample was dissolved in a solution of 94% THF, 5% DMSO and 1% piperidine, and organic phase permeation chromatography (GPC) was performed using the same solvent as an eluent at a flow rate of 1 mL/minute. The detector (Waters 2414 refractive index detector) and columns (three Waters Styragel columns, HR1, HR3, and HR4, in series) were maintained at 40°C throughout the runs. Polymer molecular weights presented are relative to monodisperse polystyrene standards (Shodex, Japan).

### Subretinal Injections

Subretinal injections were performed in both eyes of 3 month old C57Bl6 mice using a Pico-Injector (PL1-100, Harvard apparatus, Holliston, MA). Mice were anesthetized by intramuscular (IM) injection of Ketamine (80 mg/kg)/Xylazine (16 mg/kg). Pupils were dilated using 2.5% Phenylephrine Hydrochloride Ophthalmic solution (AK-DILATE, Akorn, Lake Forest, IL) followed by administration of 0.5% Tetracaine Hydrochloride Ophthalmic solution (Phoenix Pharmaceutical Inc., St. Joseph, MO) eye drops just before the injection. The conjunctiva adjacent to the cornea was grasped with forceps to allow optimal exposure of the injection site. A hole was made at the pars plana with the tip of a 30-gauge sterile (PrecisionGlide) needle. A 20∼30 µm (I.D.) micropipette glass needle tip mounted on a Pico-Injector holder was inserted in the hole, through the vitreous, and into the potential subretinal space. One µl of PBAE nanoparticles with eGFP pDNA and/or naked eGFP pDNA (at 1 mg/ml DNA, as described above in “Preparation of lyophilized nanoparticles”) was then delivered. The retinal area injected was visualized as a retinal bleb or a slight retinal detachment. Subretinal injections were made under direct observation aided by a Zeiss dissecting microscope at 6× magnification. Immediately following injection of the nanoparticles, a small amount of Bacitracin Zinc and Polymyxin B Sulfate Ophthalmic ointment (Akorn, Buffalo Grove, IL) was applied to the eye. Mice were sacrificed 72 hr later with ether and cervical dislocation. Eyes were enucleated, and the cornea and lens were removed. The retina and RPE/choroid were dissected and prepared for RNA extraction. The fellow eye was prepared for flat mount using confocal microscopy. Fluorescence intensity was analyzed with ImagePro software. The eyes with surgical complications were excluded from the study.

### Statistical Analysis

GraphPad Prism 5 software was used for all statistical analysis. For comparison of best polymer formulation (B5-S5-E7 at 60 w/w) with commercially available controls with respect to transfection and reduction in cell metabolic activity, we performed a 1-way ANOVA with a Dunnet post-test using B5-S5-E7 at 60 w/w as the control column. For comparison of base polymer structural effects with respect to transfection and reduction in cell metabolic activity, we performed a 2-way ANOVA with a Bonferroni post-test to compare all columns to each other. A two-tailed unpaired Student's t-test was used for comparison of mRNA expression after subretinal injection of nanoparticles versus pDNA alone.

## Results

*Transfection efficacy and cytotoxicity in ARPE-19 cells:* The transfection efficacy of the nanoparticle formulations (30 and 60 w/w polymer∶DNA ratio, 600 ng/well DNA dose in a 96 well plate) ranged from 0–44% GFP+ cells (**Figure 2**), with only small, single carbon changes to the backbone of the polymer structure or small changes to the ends of the polymers. To look at the effects of single carbon changes along the base polymer with respect to transfection efficacy, we averaged across the various end-modified versions of the same base polymer (**Figure 3**) and performed a 2-way ANOVA to examine the trends statistically (**Table 1**). Generally, transfection efficacy tended to increase with increasing hydrophobicity of the diacrylate or side chain in the base polymer. Interestingly, for the least hydrophobic backbones (B3b, B4) the biggest changes (and the only statistically significant ones) with respect to transfection efficacy occurred when increasing the side chain hydrocarbon length from 3 to 4 carbons, whereas with the more hydrophobic backbones (B5, B6), the only statistically significant increases in transfection efficacy occurred when increasing the side chain hydrocarbon length from 4 to 5 carbons. Only intermediately hydrophobic base diacrylates B4 and B5 showed strong statistically significant improvements when increasing the side chain length from 3 to 5 carbons.

The vast majority of the nanoparticle formulations showed only small reductions in cell metabolic activity compared to untreated controls (**Figure 4**), with the exception of some of the B6-S4, B6-S5 and E9-end modified polymers. To look at the effects of single carbon changes along the base polymer with respect to cell cytotoxicity, we averaged across the various end-modified versions of the same base polymer (**Figure 5**) and performed a 2-way ANOVA to examine the trends statistically (**Table 1**). Cytotoxicity, like transfection efficacy, also tended to increase with increasing hydrophobicity of the diacrylate or side chain in the base polymer (**Figure 5**). There was increased cytotoxicity with the most hydrophobic side chain, S5 (p<0.001 for 60 w/w with B3, B4, B5 and B6) as compared to the least hydrophobic side chain S3. Interestingly, The least hydrophobic backbones (B3, B3b) only showed increased cytotoxicity when the side chain length increased from 4 to 5 carbons and not from 3 to 4 carbons at 60 w/w, whereas the more hydrophobic backbones (B4, B5, and B6) showed increased cytotoxicity when the side chain length increased from 3 to 4 carbons (p<0.01 for all 3), but no significant increase in cytotoxicity with side chain length increasing from 4 to 5 carbons. Cytotoxicity also increased with increasing hydrophobicity of the base diacrylate, although there seems to be no increase in toxicity when the backbone contains 4 carbons (B4) between diacrylate groups as compared to only 3 (B3).

The formulation with the highest transfection efficacy was B5-S5-E7 at 60 w/w polymer∶DNA (44±5%), and it was also relatively non-toxic (23% cell cytotoxicity) following transfection. This is significantly higher transfection than has been previously reported in the literature [11], [12], [13], and was significantly higher than optimized formulations of FuGeneHD (Roche) or Lipofectamine 2000 (Invitrogen), two of the leading commercially available, cationic lipid based transfection reagents, or 25 kDa branched polyethyleneimine (**Figure 6**). Lipofectamine 2000 was optimal at a 600 ng pDNA dose with a 4 to 1 ratio of lipid∶DNA and enabled 26±7% transfection and 16±3% toxicity. X-tremeGENE HP DNA was optimal at a 600 ng DNA dose with a 5 to 1 ratio of lipid∶DNA, yielding 22±6% transfection and 32±9% toxicity. Branched 25 kDa polyethyleneimine (PEI) performed optimally at a 3∶1 ratio at 600 ng DNA/well, only achieving 8±1% transfection efficacy with 25±6% toxicity. A 1-way ANOVA with a Dunnet post-test with B5-S5-E7 as the control column showed that B5-S5-E7 at 60 w/w was significantly more efficacious than any of the positive controls (p<0.001) but maintained comparable cell viability (p>0.05 for all controls at 600 ng/well except for Lipofectamine 200 at a ratio of 2∶1).

In general, increased transfection brought with it concomitant increased cytotoxicity (**Figure 7**), but many formulations were either ineffective but moderately toxic or effective but fairly non-toxic. In general, among the base polymers which enabled high transfection, polymers containing B3-S5 were on the “bad” side of the best fit line (higher cytotoxicity relative to their transfection) and B4-S5 containing polymers were on the “good” side of the best fit line (lower cytotoxicity relative to their transfection). This can also vary by end group – B5-S5-E7 achieved the highest transfection efficacy (44±5%) with relatively low cytotoxicity (23±6%), but B5-S5-E4, with the same base polymer but different end group, transfected only 22% of cells with 39% cytotoxicity.

*Nanoparticle Physical Characterization:* Our lead polymer from the *in vitro* study, B5-S5-E7, when prepared in a large batch and purified, is moderately polydisperse, with a polydispersity index of 3.2, a number averaged molecular weight of 25,000 Da, and a weight-averaged molecular weight of 80,000 Da. It is hydrolytically degradable, with a free polymer half-life of 4.6 hr in physiological salt solution at 37°C (**Figure 8**). When complexed with pDNA, it forms nanoscale particles (hydrodynamic diameter of 180 nm), which are positively charged (zeta potential of +26 mV). The particles, when lyophilized, retain these characteristics, showing very comparable zeta potential and particle size after undergoing the freeze-drying process and being resuspended (**Figure 9**).

*Subretinal Injection of Lyophilized GFP-Nanoparticles:* A successful subretinal injection caused a bleb or retinal detachment of approximately 1/8 to 1/4 of the retina. Subretinal injection (n = 5) of 1 µl of 1 mg/ml lyophilized pDNA nanoparticles (60 w/w B5-S5-E7:DNA) resulted in significant transfection in the area of injection (**Figure 10B**), and quantitatively increased by over 1000-fold the expression of GFP mRNA by RT-PCR in both the retina (p<0.001) and the RPE/choroid (p = 0.003; **Figure 10C**) 72 hr post injection as compared to control subretinal injections of pDNA alone (n = 5 for nanoparticle groups, n = 3 for controls). In an absolute sense, nanoparticle injection increased GFP mRNA transcript levels to about ½ that of GAPDH. Intensity data from florescence micrographs (n = 3) showed increased fluorescence in 2 of 3 of the RPE flat mounts which were exposed to the nanoparticles, but in aggregate the difference was not statistically significant compared to injection of pDNA alone (p = 0.08; **Figure 10D**).

## Discussion

Standard non-viral transfection protocols for *in vitro* transfection of RPE cells achieve only limited to moderate success in terms of transfection efficiency. In addition, unlike typically optimal *in vitro* transfection conditions, where the transfection is done without serum and with cells in a sub-confluent state to maximize transfection, *in vitro* conditions were chosen here to more closely match the *in vivo* state. As RPE cells in the mature eye are not rapidly dividing and particles will come into contact with proteins before reaching cell surfaces, we used confluent cells in the presence of serum for our *in vitro* studies.

Figures 2–6 show interesting trends to guide the design of polymeric nanoparticles for transfection of retinal pigment epithelial cells. As seen previously in studies looking at transfection of COS-7 monkey kidney cancer cells [17], we can see interplay between the hydrophobicity of the base diacrylates and the side chain amino-alcohols (for statistical analysis, see **Table 1**). For example, relatively hydrophilic base polymers containing the shortest side chain, S3, were generally ineffective, and required the most hydrophobic base diacrylates, B5 and B6, to see any transfection efficacy. They were also non-toxic. On the other hand, polymers containing the most hydrophobic side chain, S5, were nearly all reasonably effective in transfection. Optimal transfection for many end-groups was achieved with the intermediately-hydrophobic base-diacrylates B4 and B5 and most hydrophobic side chain S5. End-modified polymers with the most hydrophobic base group (B6) did not tend to be as effective and also were associated with more cytotoxicity, and B3-S5 end-modified polymers were not as efficacious but were non-toxic. Interestingly, increasing the hydrophobicity of the side chain from 3 to 4 carbons in length tended to increase the transfection efficacy of polymers containing the least hydrophobic base diacrylates without increasing the cytotoxicity, whereas increasing the side chain length from 4 carbons to 5 carbons for the most hydrophobic base diacrylate only increased the cytotoxicity of the formulation without increasing transfection efficacy. Taking this together suggests that polymer hydrophobicity plays a key role in the transfection efficacy of these complexes, but that there is a limit at which polymer hydrophobicity no longer increases transfection efficacy as fast as it increases cytotoxicity, such that there is an optimal hydrophobicity for PBAE based non-viral gene delivery vectors.

End-modification of the polymers had a clear and strong effect, with polymers end-modified with E1 and E3-E8 tending to be more successful, and polymers modified with E9, E10, and E12 end-modifications tending to be less effective. Increasing polymer to pDNA weight ratio from 30 w/w to 60 w/w tended to both increase transfection efficacy and cytotoxicity; however, even at 60 w/w the cytotoxicity was moderate for most transfection conditions. Molecular weight was previously found to not vary the transfection efficacy of these materials [17], so that these differences in efficacy are likely due directly to the small changes in chemical structure that we observe. These small changes in structure may play a role in changing the DNA/polymer binding properties, changing the particle size, and changing the degradation rate of the nanoparticles, all of which could play a significant role in the observed gene delivery properties of the polymer library.

We next sought to test the most successful type of nanoparticle *in vivo*. However, *in vivo* injections into the eye require low volumes and highly concentrated pDNA nanoparticles. The maximum volume that can be injected into the mouse eye is 1 µL. Simply scaling down our transfection protocol to the 1 µl volume required for the subretinal injections would have allowed us to deliver only 30 ng of DNA to the eye. Due to the hydrophobicity of the polymers, formulation of particles at greater than 30-fold higher concentration (which would be required for our desired 1 µg dose to the eye) in standard aqueous conditions is not possible due to polymer solubility constraints. However, our lab has recently developed a protocol to formulate PBAE-based nanoparticles by adding sucrose as a cryoprotectant and then freeze-drying the particles. This process has been shown to allow long-term storage of the particles, with complete retention of transfection efficiency in glioblastoma cells after 3 months [18]. Although *in vitro* transfection efficacy has been validated using this procedure, *in vivo* efficacy of these lyophilized PBAE nanoparticles is unknown. Since the dry polymer/DNA nanoparticles can be resuspended following lyophilization in minimal water volumes, we hypothesized that we could increase nanoparticle concentration using this procedure. This is likely because resuspension of the nanoparticles requires formation of a colloidal suspension of polymer/DNA nanoparticles and does not require solvation of free polymer at the same concentrations. The cryoprotectant is also required for this step, as particles formed without cryoprotectant were unable to be resuspended. We were able to formulate 60 w/w particles with 30 µg of pDNA, add sucrose, freeze dry the particles, and then resuspend the particles to 30 µl total volume in water, thus allowing the injection of 1 µg of DNA in 1 µl. This technique has several advantages beyond simply increasing the concentration of the particles, such as easy storage and low risk of degradation.

Significant transfection of the RPE and retina was achieved with subretinal injection of 1 µl of 1 mg/ml DNA nanoparticles, in the area of injection (**Figure 9B**). Quantitatively, there was over 1000-fold increase in expression of GFP mRNA by RT-PCR in both the retina and the RPE/choroid (**Figure 9C**). The relative expression of GFP mRNA varied between the different mice (86–3800 fold increase in the retina, and 50–5000 fold increase in the RPE/choroid), compared to naked eGFP DNA injection. Nonetheless even the lowest transfection is significantly higher than other delivery systems. A recent article by Muna Naash's laboratory studied CK30PEG10k complexed with eGFP to transfect RPE and found a 2.5 fold increase in eGFP expression in the RPE compared to a naked pDNA control [23]. Interestingly, they found similar uptake of GFP with CK30PEG10k as with naked GFP due to the RPE cell's ability to phagocytoze naked GFP, and attributed their 2.5-fold improvement in transfection to improvements in downstream nuclear import [23]. Here, we are able to show a >1000-fold increase in GFP expression in the retina and RPE with our nanoparticles, which are specifically designed for improved intracellular delivery. PBAE nanoparticles are believed to improve intracellular delivery by binding and protecting pDNA, and facilitating both endosomal escape and the release of the DNA to the cytoplasm [20].

Here we show that poly(beta-amino) ester-based nanoparticles have great promise for delivery of plasmids to RPE cells. These nanoparticles are small in size (180 nm), have a positive zeta potential (+26 mV), and easily degrade in water. Many polymer formulations showed transfection efficacy that was significantly superior to Lipofectamine 2000 and FuGeneHD, two of the lead commercially available alternatives for non-viral gene delivery, with comparable cellular viability. The lead polymer, 1-(3-aminopropyl)-4-methylpiperazine-end-modified poly(1,5 pentanediol diacrylate-co-5-amino-1-pentanol) (B5-S5-E7), at 60 w/w polymer∶DNA ratio was able *in vitro* to transfect 44% of ARPE-19 cells that were visually confluent with minimal cytotoxicity. Therefore, this novel nanoparticle has many potential benefits to further investigate ocular diseases. The successful transfection of the RPE *in vivo* with lyophilized nanoparticles using this polymer formulation suggests that this technology could be useful in the future *in vivo* both in the lab and possibly in the clinic, to help ameliorate genetic diseases of the retina.
